# The frequency and availability of population-specific patient reported outcome measures and minimal clinically important differences among approved drugs in Canada

**DOI:** 10.1186/s12955-018-1070-0

**Published:** 2019-01-07

**Authors:** Allison Soprovich, Meghan Ingstrup, Dean T. Eurich

**Affiliations:** Alliance for Canadian Health Outcomes Research in Diabetes, School of Public Health, University of Alberta, Edmonton, Alberta T6G 2E1 Canada

**Keywords:** Patient reported outcome measures, Minimal clinically important difference, Health technology assessment, Transparency

## Abstract

**Background:**

Patient reported outcome measures (PROMs) and minimal clinically important differences (MCIDs) are included in Canada’s Common Drug Review (CDR) process to approve new drugs. Often, the measures report on the health-related quality of life (HRQoL), but can also describe the symptoms, efficacy and harms important to patients. They can be generic or population/condition specific, validated or not. We examined the frequency, availability and accessibility of validated, specific PROMs and MCIDs reported in the CDR reports.

**Methods:**

We searched the Canadian Agency for Drugs and Technologies in Health (CADTH) on-line database for completed Common Drug Review, Clinical Review Reports (CDR-CRR) between November 2013 and February 2017. Two independent reviewers examined the reports and references for PROMs and MCIDs. Both reviewers separately categorized the PROMs and MICDs according to purpose, validation, availability and funding received. Discrepancies were rectified by consensus with a third investigator.

**Results:**

One-hundred and five unique PROMs were extracted from 39 CDR-CRR, 57% with a HRQoL component. 91/105 (87%) referenced a validation study and 62/105 (59%) referenced a validation study in the study population of interest. Fifty-seven MCID references were extracted from 39 CDR-CRR. 34/57 (60%) were specific to the study population of interest, and 36% had a HRQoL component. 50% of PROM and 53% of MCID references were publicly available.

**Conclusions:**

PROMs and MCIDs referenced in CDR-CRR show similar trends. The majority are validated, but not necessarily in the study population of interest. Continued critical examination is required to evaluate new drugs specific to the population of interest.

**Electronic supplementary material:**

The online version of this article (10.1186/s12955-018-1070-0) contains supplementary material, which is available to authorized users.

## Background

Since 2010, the Canadian Agency for Drugs and Technologies in Health (CADTH) has incorporated patient input into their review process for drug coverage, though not mandatory [1]. Patients have the opportunity to comment on the efficacy, harms, health-related quality of life and cost associated with providing public coverage for these drugs. Patients provide input through an organized patient group, if one exists, by completing a standardized template describing the disease experience, experiences with currently available treatments, improved outcomes, experience with the drug under review and companion diagnostic testing. Calls for patient input are posted on the CADTH website and distributed through CADTH E-Alerts, giving patients 35 business days to prepare and submit. Many of the clinical trials comprising the drug reviews also include Patient Reported Outcome Measures (PROMs) that describe patient perspectives on treatment and disease impacts. They aim to capture the personal and social context of the disease and treatment experiences to assess the full impact of a treatment [2]. These measures are helping to move patient centered practices forward.

Another evolution of patient involvement in clinical care and research, is the development of Minimal Clinically Important Differences (MCIDs). This score is defined as the minimal amount of change that is important to the patient [3], and an important concept when evaluating the real-world value of a drug or treatment. A study may show statistical significance, but this may not have the treatment impact patients’ value; and vice versa. The MCID is not a fixed attribute, rather it is specific to a disease state and population [3]. This makes it difficult to measure, but provides very specific information relating to outcomes that matter to patients.

Patient-centered care, shared decision making and evidence based medicine are becoming widely used practices; however, they require objective, unbiased research to be publicly accessible [4]. Wieseler et al. [5] found a substantial number of patient-relevant outcomes were missing from the public record, making unbiased trial evaluation challenging. Although organizations like CADTH have full access to clinical study reports and data to appraise the drugs, public access to other patient-relevant outcomes may be limited. Open access journals aim to provide unrestricted online access to scholarly publications [6]. One study compared open access articles to non-open access articles and found open access articles to be twice as likely to be cited 4–10 months after publication, and almost three times as likely to be cited 10–16 months after publication [7]. This type of access helps promote the acceleration of dissemination and uptake of results [7]. If we are to advance in patient involvement in clinical care, regular patients and clinicians will need to have timely access to the research findings.

In response to these three patient-related issues, we examined the incorporation of PROMs, MCIDs and public access in the CADTH Common Drug Review, Clinical Review Reports (CDR-CRR). Our aims were 1) to measure the frequency of PROM reporting in CDR-CRR; 2) report if the PROMs are validated in the study population with a corresponding MCID; and, 3) measure the frequency of public availability and accessibility of the PROM/MCID reference.

## Methods

We searched the CADTH on-line database for completed CDR-CRR between November 2013 and February 2017. Drugs with a completed CDR-CRR between those dates were included. Two independent reviewers performed a critical examination of the selected reports for PROMs and MCIDs. PROMs were defined as referenced instruments used to directly report patient response without interpretation by a clinician or anyone else [8]. We included any measurement with a patient-reported (or proxy-reported) component. The documented PROMs were sub-sequentially categorized as symptom, treatment, adverse events or HRQoL measures. Both single dimension and multi-dimension measures, and generic and disease-specific measures were included. Those disease-specific PROMS with multiple components (i.e. both symptoms and treatment) were dually classified. Discrepancies were discussed with a third investigator until consensus was reached.

MCIDs were defined as “the smallest difference in score in the domain of interest which patients perceive as beneficial and which would yield (outside of adverse events and cost) a change in management” [9]. MCIDs were documented as either general to the PROM or else specific to the study population of interest in the CDR-CRR.

Sub-group analyses described two drug characteristics. Specialty status was defined as high cost (>$500/dose or $6000/year), high complexity (physician specialist involvement and/or administration), and/or high touch (cold chain maintenance). Any product with biologic components was coded as biological agent. These definitions have been previously published [4].

The public availability of references of the PROM or MCID included in the CDR-CRR were also examined and documented by two independent reviewers, and classified as publicly available, restricted access (i.e. payment or log-in required), or not available. This was performed using public internet access in a residential setting in Alberta, Canada. References were also reported as industry funded, if stated to have received any industry funding.

Basic descriptive analysis was completed with Microsoft Excel and STATA MP (version 14.12). The total number of PROMs and MCIDs referenced were reported, excluding duplicate PROMs found in the same study population (e.g. EQ-5D used in adults with hepatitis C in two different CDR-CRRs). Duplicates were found in chronic hepatitis C, juvenile arthritis, overactive bladder, macular edema and adults with rheumatoid arthritis populations. We also extracted those PROMs with any HRQoL component as a sub-group. We documented both general validation references as well as those specific to the study population of interest in the CDR-CRR. The drugs evaluated in the CDR-CRRs were described according to specialty status, biologic components and funding received, as defined above.

## Results

We found 62 CDR-CRR completed between November 1, 2013 and February 16, 2017. 39 were included in the analysis; 18 (29%) did not report any PROM or HRQoL measurement, 4 (6.5%) reported duplicate PROMs in the same study population and 1 (1.6%) was not specific enough. 142 PROMs were reviewed; 105 unique PROMs within a unique study population were extracted and included in the analysis (see Additional file 1 for exclusions and justifications, Additional file 2 for included drugs and PROMs).

60/105 (57%) PROMs had a HRQoL component and the majority (59%) were disease specific. Neurological (20%) and rheumatological disorders (13%) were the most common conditions reporting disease specific PROMs.

**Fig. 1:**
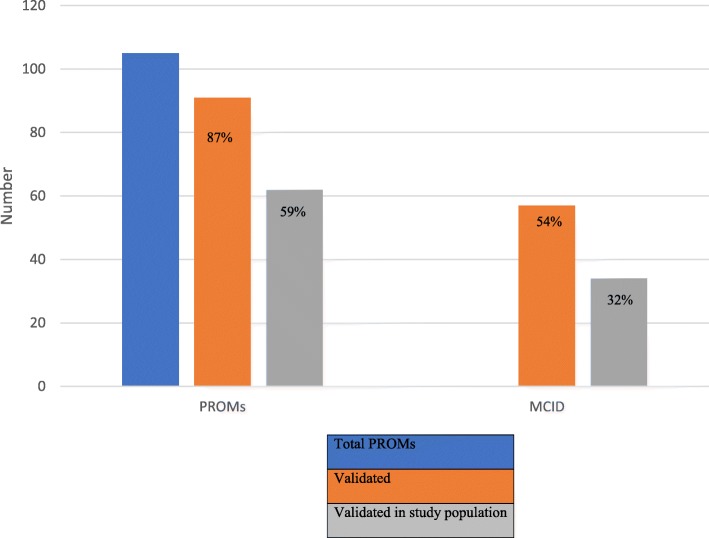
– Frequency of PROMs and MCID reported

**Fig. 2:**
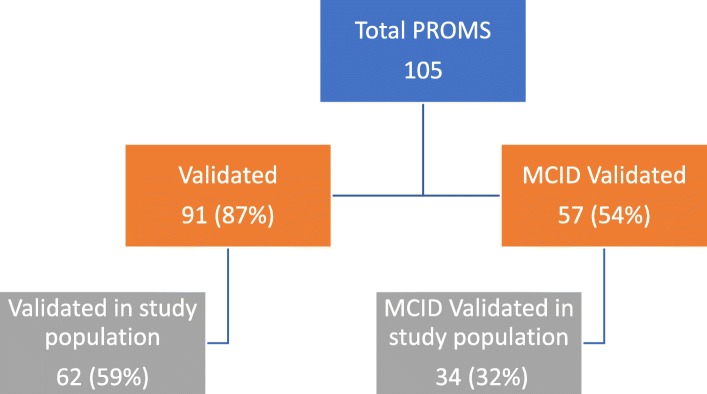
Flow diagram of validation of PROMs and MCID

Our primary analysis explored the validation frequency of PROMs referenced in CDR-CRRs (Fig. 1). Of the 105 total PROMs, 91 (87%) referenced a validation study, and 62 (59%) referenced validation in the study population of interest in the CDR-CRR (Fig. 2). Of the 60 PROMs with a HRQoL component, 55 (92%) referenced a validation study and 36 (60%) referenced validation in the study population of interest. 69% of the time, PROMs validated in the study population were in specialty drugs (*p* = 0.021) where CDR-CRRs including biologic agents were less likely to be validated (60%, *p* = 0.586).

We found 57 of 105 PROMS (54%) contained at least one MCID reference. 32% (34/105) of PROMs had a MCID specific to the study population of interest in the CDR-CRR (Fig. 2). 34/57 (60%) of MCIDs were specific to the study population. 67% (38/57) of MCIDs and 59% (20/34) of the specific study population MCIDs had a HRQoL component. Neurological and respiratory conditions reported a MCID most frequently, 75 and 88% of the PROMs respectively.

**Fig. 3:**
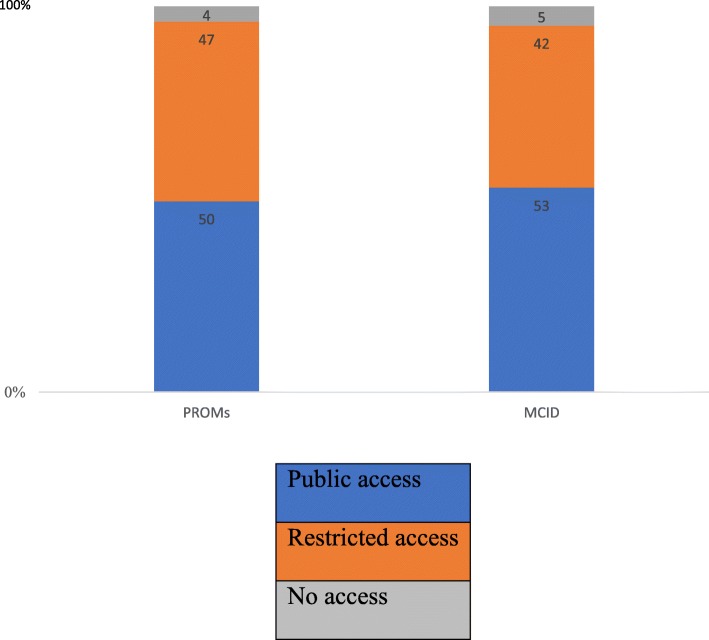
Percentage according to access type – study population validation references

The 62 PROMs validated in the study population comprised of 111 references. Our secondary analysis explored the public availability of references used in the CDR-CRRs (Fig. 3). We found 55/111 (50%) PROM validation references specific to the study population of interest were publicly available; 52/111 (47%) had restricted access; and 4/111 (4%) with no access. 48/111 (43%) specified industry funding received in their development.

The 34 MCIDs specific to the study population of interest comprised 43 references. With respect to MCID specific to the study population of interest, we found 23/43 (53%) of MCID references were publicly available; 18/43 (42%) had restricted access; and 2/43 (5%) with no access. 26/43 (60%) specified industry funding received.

When comparing those study population validated PROMs with a HRQoL component to those without, no significance was found (*p* = 0.910) between groups (Table 1). When comparing disease specific to generic PROMs, only the specific study population validation reference was significant for PROMs (*p* = < 0.001) and MCIDs (*p* = < 0.001) (Tables 2 and 3).

**Table 1:** Study population validation reference comparison of PROMs with a HRQoL component to those without

Validation of PROM	HRQL PROM n (%)	Non-HRQL measure n (%)	Total n (%)
Not validated	20 (33)	15 (33)	35
Validated^a^	36 (60)	26 (58)	62
No reference given	4 (7)	4 (9)	8
Total	60	45	105 (*p* = 0.910)

^a^Validation not evaluated

**Table 2:** Study population validation reference comparing disease specific to generic PROMs

PROM validated in study population	Disease specific n (%)	Generic n (%)	Total n (%)
Not validated	11 (18)	24 (56)	35
Validated^a^	47 (76)	15 (35)	62
No reference given	4 (6)	4 (9)	8
Total	62	43	105 (*p* = < 0.001)

^a^Validation not evaluated

**Table 3:** MCID references comparing disease specific to generic PROMs

MCID reported	Disease specific n (%)	Generic n (%)	Total n (%)
No MCID	32 (55)	33 (80)	65 (66)
MCID	26 (45)	8 (20)	34 (34)
Total	58	41	99 (*p* = 0.009)

## Discussion

We found 105 PROMs and 57 MCID references reported in 39 CDR-CRR. Though these PROMs and MCIDs are not always validated in the study population, a large majority were. Similar trends in both PROM and MCID reporting regarding frequency and public access were observed. 87% of PROMs had a validation reference; 59% validated in the study population and 60% of MCIDs reported were also validated in the study population.

Although 29% of all CDR-CRRs did not report any PROM or HRQoL measurement, when included, multiple PROMs were used for the same drug and/or trial, each with its’ own purpose. It is challenging to choose the right PROM when designing a study, as patients can provide details on a number of domains that are important for evaluation, such as symptom experience, functional status, well-being, quality of life and treatment satisfaction [10]. When designing a study, a number of relevant existing PROM measures could exist or none may seem appropriate. Furthermore, a drug may not be expected to affect all domains equally within a PROM. Often, there is overlap among generic PROMs with questions that could seem redundant to patients, affecting response and attrition rates.

Recently, the International Society for Quality of Life Research (ISOQOL) conducted a literature review and accompanying survey of its members to identify minimum standards for the design and selection of a PROM for use in patient-centered outcomes research and comparative effectiveness research. Evidence for reliability, validity (content, construct and responsiveness), interpretability of scores, quality translation and acceptable patient and investigator burden were the final recommendations for standards and considerations [10]. They emphasized the important of availability of the documentation of the PROM evidence to generate greater acceptance and use of the measure [10].

As policy concerns become more focused with research uptake and knowledge translation, the issue of interpretability of scores surfaces. MCID were developed, originally promised to define improvement thresholds, helping to interpret results and guide clinical care. However, some remain skeptical about reaching this type of objectivity. We found 60% of MCID references included in the CDR-CRRs were specific to the study population, which reflects the context-specific nature of the MCID. MCID variability depends on baseline scores, study context and the approach used to determine the MCID [11], time between baseline and follow up [12], and may vary according to the direction of the score. In patient management, MCID help clinicians interpret and guide clinical practice in a systematic way. It can enhance patient-provider and provider-provider communication [13]. Beaton et al. conclude that future work on MCID should determine whether or not a MCID for improvement is the same as that for deterioration [11] within the same context. The MCID plays a crucial role in determining any level of change and responsiveness [12]. It is extremely useful in clinical research, where high powered studies will often report statistically significant results due to the low type 2 errors, without measuring clinical significance [14]. Here, reviewers and policy makers need to acknowledge clinical significance and the situation-specific nature of the MCID, and consider this carefully when making final recommendations.

The goal of any research is for it to be used, applied and expanded upon. Public accessibility is important for the timely dissemination of new research to clinicians, patients and the public. The concept of Open Access increases the potential readership of any article to over a billion individuals with Internet access and indirectly speeds up the spread of information [6]. With increased focus on patients and their families through the use of PROMs, it only makes sense to have these findings available to those who contributed, will use and will further discuss the results in the community. With more accessible PROM and MCID data and reports, it can also influence clinical decision making and policy. Future directions might consider evaluating whether or not patients actually access and read research and how much impact more technical validation studies and publications have to patients. Taking this one step further, we might consider alternative knowledge translation products, keeping in mind the lay person audience, and how the research results can be understood and used by those who contributed to the research in the first place.

Patients have the opportunity to contribute to the current CADTH review process through a patient input section. This is almost always narrative accounts, with very little real-world data. This paper adds emphasis to the increasing number of PROMs included in drug studies and reviews, with a focus on public accessibility of data and reports. Patients are a knowledgeable group, with lived experience. With the right education and organization, patients may be able to collect their own PROMs to allow for greater comparisons across clinical trial and real-world data, giving strength to the patient input section of the CADTH drug review. However, first, we must continue to value PROMs and MCID research at large.

### Limitations

Our review is not without limitations. Though we examined 105 PROM measurements, these were found in only 39 CDR-CRR reports and this sample size limited our analyses. CDR-CRRs are also released in ‘batches’ and it’s possible some reports were not yet publicly available to be included. As well, we could not control for the types of diseases or conditions for which CDR-CRR reports were generated. We were unable to identify which fields or diseases lack PROM and MCID studies. Additionally, academic researchers determined the public availability of the references and although double data entry was performed from public locations, access may have been granted due to affiliation. The public availability of research varies greatly around the world and our findings may not be accurate for other jurisdictions.

## Conclusions

We found similar trends when examining the frequency and public availability of PROM and MCID references in CADTH’s CDR-CRR. Most PROMs had a validation reference with the majority reported in the study population of interest. Only (57/105) 54% of PROMs reported an MCID, resulting in 32% of PROMs reporting a population-specific MCID. The majority of PROM and MCID references were publicly available. Future research will likely validate more PROMs and MCIDs, however, the continued critical examination of these features of new drug approvals is necessary to push patient-centered care and evidence-based medicine forward, allowing patients to both express themselves and access the information.

## Additional files

Additional file 1: **Table 4.** Exclusions and justifications. A complete listing of all drugs excluded from analysis and justifications (DOCX 17 kb)

Additional file 2: **Table 5.** Included drugs and PROMs. A complete listing of all drugs and PROMs included in the analysis (DOCX 22 kb)

## Abbreviations

CADTH: Canadian Agency for Drugs and Technologies in Health
CDR-CRR: Common Drug Review, Clinical Review Reports
HRQoL: Health-related quality of life
ISOQOL: International Society for Quality of Life Research
MCID: Minimal clinically important differences
PROM: Patient reported outcome measure
