# Supplementary material for: The frequency and availability of population-specific patient reported outcome measures and minimal clinically important differences among approved drugs in Canada
Source: Health Qual Life Outcomes. 2019 Jan 7;17:4. doi: 10.1186/s12955-018-1070-0 (PMC6322322; doi:10.1186/s12955-018-1070-0)
Supplement: Supplementary file 1 — Table 4. Exclusions and justifications. A complete listing of all drugs excluded from analysis and justifications (DOCX 17 kb) [file 12955_2018_1070_MOESM1_ESM.docx]

Table 4 - Exclusions and Justifications

| Drug Name | Generic Name | PROM | Justification |
| --- | --- | --- | --- |
| Genotropin – Adult | somatropin | Multiple PROMs used | Not specific enough |
| Genotropin – TS | somatropin | “evaluated psychological aspects” | Not specific enough |
| Genotropin – Pediatric | somatropin | None reported |  |
| Latuda | lurasidone | None reported |  |
| Xarelto | rivaroxaban | None reported |  |
| Inspra | eplerenone | None reported |  |
| Aubagio | teriflunomide | Multiple sclerosis functional composite | Not directly patient reported |
| Komboglyze | saxagliptin + metformin | None reported |  |
| Grastek | phleum pratense | Daily medication score | Objective outcome measurement data |
| Diacomit | stiripentol | None reported |  |
| Picato – AK | ingenol mebutate | None reported |  |
| Invokana | canagliflozin | None reported |  |
| Kazano | alogliptin + metformin | None reported |  |
| Nesina | alogliptin | None reported |  |
| Prezcobix | darunavir/cobicistat | None reported |  |
| Juxtapid | lomitapide | None reported |  |
| Triumeq | dolutegravir/abacavir/lamivudine | None reported |  |
| Eliquis | apixaban | None reported |  |
| Simbrinza | brinzolamide/brimonidine | None reported |  |
| Prolia - Men | denosumab | None reported |  |
| Naglazyme | galsulfase | None reported |  |
| Grastofil | filgrastim | None reported |  |
| Myrbetriq | mirabegron | King’s Health Questionnaire | Duplicate PROM |
| Inflectra | infliximab | ACR 20/50/70 | Duplicate PROM |
| Inflectra | infliximab | SF-36 | Duplicate PROM |
| Actemra – A,R | tocilizumab | ACR 20/50/70 | Duplicate PROM |
| Actemra – A,R | tocilizumab | Health Assessment Questionnaire | Duplicate PROM |
| Actemra – A,R | tocilizumab | SF-36 | Duplicate PROM |
| Harvoni | ledipasvir/sofosbuvir | Chronic Liver Disease Questionnaire-HCV | Duplicate PROM |
| Harvoni | ledipsavir/sofosbuvir | Functional Assessment of Chronic Illness Therapy - Fatigue | Duplicate PROM |
| Harvoni | ledipasvir/sofosbuvir | SF-36 | Duplicate PROM |
| Harvoni | ledipsavir/sofosbuvir | Work Productivity and Activity Impairment – Hep C | Duplicate PROM |
| Revolade | eltrombopag | Chronic Liver Disease Questionnaire - HCV | Duplicate PROM |
| Eylea – CRVO | afibercept | National Eye Institute 25-item Visual Function Questionniare | Duplicate PROM |
| Eylea – CRVO | afibercept | EQ-5D | Duplicate PROM |
| Ilaris | canakinumab | ACR 20/50/70 Pediatric | Duplicate PROM |
| Ilaris | canakinumab | Childhood Health Assessment Questionnaire | Duplicate PROM |
