# Supplementary material for: The frequency and availability of population-specific patient reported outcome measures and minimal clinically important differences among approved drugs in Canada
Source: Health Qual Life Outcomes. 2019 Jan 7;17:4. doi: 10.1186/s12955-018-1070-0 (PMC6322322; doi:10.1186/s12955-018-1070-0)
Supplement: Supplementary file 2 — Table 5. Included drugs and PROMs. A complete listing of all drugs and PROMs included in the analysis (DOCX 22 kb) [file 12955_2018_1070_MOESM2_ESM.docx]

Table 5 - Included drugs and PROMs

| Drug Name | Generic Name | PROM/HRQL scale used |
| --- | --- | --- |
| n=39 |  | n=105 |
| Fibristal | ulipristal acetate | Pictorial Bleeding Assessment Chart |
|  |  | SFMPQ (Short-form McGill Pain Questionnaire) |
|  |  | UFS-QoL (Uterine Fibroid Symptom and Health-Related Quality of Life Questionnaire) |
|  |  | measurement of discomfort questionnaire |
| Onglyza | saxagliptin | EQ-5D |
| Genotropin - Adult | somatropin | QoL-AGHDA (Quality of Life - Assessment of Growth Hormone Defiiciency in Adults) |
|  |  | NHP (Nottingham Health Profile) |
|  |  | PGWBI (Psychological General Well-Being Index) |
| Jetrea | ocriplasmin | NEI-VFQ (National Eye Institute Visual Function Questionniare) |
| Picato - KA | ingenol mebutate | Skindex-16 |
|  |  | TSQM (Treatment satisfaction questionnaire for medication) |
| Actemra - JA | tocilizumab | ACR Pedi (American College of Rheumatology pediatric criteria) |
|  |  | CHAQ (Childhood Health Assessment Questionnaire) |
| Simponi | golimumab | Mayo Scoring System |
|  |  | IBDQ (Inflammatory Bowel Disease Questionnaire) |
| Tudorza Genuair | aclidinium bromide | EXACT-PRO (Exacerbation of Chronic Pulmonary Disease tool - Patient-reported Outcomes |
|  |  | SGRQ (St. George's Respiratory Questionnaire) |
|  |  | Baseline Dyspnea Index (BDI)/Transition Dyspnea Index(TDI) |
|  |  | EQ-5D |
| Botox - MC | onabotulinumtoxin A | Migraine-Specific Quality of Life Questionnaire (MSQ) |
|  |  | Headache Impact Test (HIT) |
| Neupro | rotigotine | Unified Parkinson's Disease Rating Scale |
|  |  | PDQ-39 (Parkinson's Disease Questionnaire-39) |
|  |  | EQ-5D |
|  |  | Parkinson Home Diary |
|  |  | Parkinson's Disease Sleep Scale |
| Aubagio | teriflunomide | Expanded Disability Status Scale |
|  |  | EQ-5D |
|  |  | Fatigue Impact Scale |
|  |  | SF-36 |
|  |  | MSQOL - 54 (multiple sclerosis quality of life) |
|  |  | Treatment Satisfaction Questionnaire for Medication |
| Galexos | simeprevir | EQ-5D |
| Adempas | riociguat | EQ-5D |
|  |  | Living with PH questionnaire |
| Simponi I.V. | golimumab | ACR 20/50 (American College of Rheumatology Response Criteria) |
|  |  | HAQ (Health Assessment Questionnaire, HAQ Disability Index) |
|  |  | SF-36 |
| Sovaldi | sofosbuvir | CLDQ (Chronic Liver Disease Questionnaire) - HVC |
|  |  | FACT (Functional Assessment of Chronic Illness Therapy - Fatigue) |
|  |  | SF-36 |
|  |  | WPAI (Work Productivity and Activity Impairment) - HepC |
| Grastek | phleum pratense | Rhinoconjunctivitis Daily Symptom Score |
|  |  | Combined Score or Total Combined Score |
|  |  | VAS (Visual Analogue Scale) |
|  |  | RQLQ (Rhinoconjunctivitis Quality of Life Questionnaire) |
| Intuniv XR | guanfacine hydrochloride | ADHD Rating Scale IV |
|  |  | Conner's Rating Scales (Teacher Rating Scale-Revised, Parent Rating Scale-Revised) |
|  |  | CHQ (Child Health Questionnaire) |
|  |  | Weiss Functional Impairment Rating Scale - Parent Report |
|  |  | Health Utility Index Mark 2 and Mark 3 |
| Eylea - Age MD | afibercept | NEI-VFQ (National Eye Institute Visual Function Questionniare) |
| Stelara | ustekinumab | American College of Rheumatology 20/50/70 |
|  |  | Psoriatic Arthritis Response Criteria |
|  |  | Disease Activity Score 28 and C-reactive protein |
|  |  | Psoriasis Area Severity Index |
|  |  | HAQ (Health Assessment Questionnaire) |
|  |  | SF-36 |
|  |  | Bath Ankylosing Spondylitis Disease Activity Index |
| Abilify | aripiprazole | Inventory of depressive symptomatology - self report (quick inventory of depressive symptomatology - self-report) |
|  |  | Sheehan Disability Scale |
|  |  | Quality of Life Enjoyment and Satisfaction Questionnaire |
| Botox - UI | onabotulinumtoxin A | IQoL (Incontinence Quality of Life Questionnaire) |
|  |  | King's Health Questionnaire |
|  |  | SF-12 |
|  |  | EQ-5D |
| Myrbetriq | mirabegron | OAB-q (Overactive Bladder Questionnaire) |
|  |  | PPBC (Patient Perception of Bladder Condition) |
| Inflectra | infliximab | Disease Activity Score 28 and European League Against Rheumatism (EULAR) response criteria |
| Kalydeco - CFTR | ivacaftor | Cystic Fibrosis Questionnaire - Revised |
| Opsumit | macitentan | Borg Dyspnea Index |
|  |  | SF-36 |
| Lucentis | ranibizumab | NEI-VFQ (National Eye Institute Visual Function Questionniare) |
|  |  | EQ-5D |
|  |  | WPAI (Work Productivity and Activity Impairment) |
| Signifor | pasireotide diaspartate | Cushing Quality-of-life Questionnaire |
| Vimizim | elosulfase alfa | MPS Health Assessment Questionnaire |
| Aptiom | eslicarbrazepine acetate | Seizure Severity Questionnaire |
|  |  | QOLIE-31 (31-item Quality of Life in Epilepsy Inventory) |
|  |  | CGI (Clinical Global ImpressionI) |
| Zaxine | rifaximin | Chronic Liver Disease Questionnaire |
| Eylea - MED | afibercept | NEI VFQ-25 (National Eye Institute 25-item Visual Function Questionnaire) |
|  |  | EQ-5D |
| Xolair | omalizumab | Urticaria Activity Score |
|  |  | Dermatology Life Quality Index |
|  |  | Chronic Urticaria Quality of Life Questionnaire |
|  |  | EQ-5D |
| Kalydeco - R117H | ivacaftor | Cystic Fibrosis Questionnaire - Revised |
| Genvoya | elvitegravir/cobicistat/emtricitabine/tenofovir alafenamide | EQ-5D |
|  |  | SF-36 |
| Basaglar | insulin glargine | Insulin Treatment Satisfaction Questionnaire (ITSQ) |
|  |  | Adult Low Blood Sugar Survey |
| Nucala | mpolizumab | SGRQ (St. George's Respiratory Questionnaire) |
|  |  | Asthma Control Questionnaire 5 |
|  |  | EQ-5D |
|  |  | SGRQ mapping to the EQ-5D utilities |
| Ilaris | canakinumab | CHQ (Childhood Health Questionnaire) |
| Entyvio | vedolizumab | Crohn's Diseae Activity Index |
|  |  | Inflammatory Bowel Disease Questionnaire |
|  |  | SF-36 |
|  |  | EQ-5D |
| Brivlera | brivaracetam | QOLIE-31-P (Patient-Weighted Quality of Life in Epilepsy Inventory-31) |
|  |  | Hospital Anxiety and Depression Scale (HADS) |
|  |  | P-GES (Patient Global Evaluation Scale) |
|  |  | EQ-5D |
